# DDX10 promotes the proliferation and metastasis of colorectal cancer cells via splicing RPL35

**DOI:** 10.1186/s12935-022-02478-1

**Published:** 2022-02-02

**Authors:** Xin Zhou, Zhihong Liu, Tengfei He, Cuifeng Zhang, Manman Jiang, Yuxiao Jin, Ziyu Wu, Changji Gu, Wei Zhang, Xiaodong Yang

**Affiliations:** 1 Department of General Surgery, The Second Affiliated Hospital of Soochow University, 1055 Sanxiang Road, Suzhou, 215004 Jiangsu China; 2 Soochow University, 1 Shizi Street, Suzhou, China; 3 Department of Radiotherapy, The Second Affiliated Hospital of Soochow University, 1055 Sanxiang Road, Suzhou, 215004 Jiangsu China

**Keywords:** Colorectal cancer, DEAD-box helicase 10 (DDX10), Oncogene, RPL35, mRNA splicing

## Abstract

**Background:**

Colorectal cancer (CRC) has become the second deadliest cancer in the world and severely threatens human health. An increasing number of studies have focused on the role of the RNA helicase DEAD-box (DDX) family in CRC. However, the mechanism of DDX10 in CRC has not been elucidated.

**Methods:**

In our study, we analysed the expression data of CRC samples from the Gene Expression Omnibus (GEO) and The Cancer Genome Atlas (TCGA) databases. Subsequently, we performed cytological experiments and animal experiments to explore the role of DDX10 in CRC cells. Furthermore, we performed Gene Ontology (GO)/Kyoto Encyclopedia of Genes and Genomes (KEGG) enrichment analysis and protein–protein interaction (PPI) network analyses. Finally, we predicted the interacting protein of DDX10 by LC–MS/MS and verified it by coimmunoprecipitation (Co-IP) and qPCR.

**Results:**

In the present study, we identified that DDX10 mRNA was extremely highly expressed in CRC tissues compared with normal colon tissues in the TCGA and GEO databases. The protein expression of DDX10 was measured by immunochemistry (IHC) in 17 CRC patients. The biological roles of DDX10 were explored via cell and molecular biology experiments in vitro and in vivo and cell cycle assays. We found that DDX10 knockdown markedly reduced CRC cell proliferation, migration and invasion. Then, we constructed a PPI network with the Search Tool for the Retrieval of Interacting Genes/Proteins (STRING). GO and KEGG enrichment analysis and gene set enrichment analysis (GSEA) showed that DDX10 was closely related to RNA splicing and E2F targets. Using LC–MS/MS and Co-IP assays, we discovered that RPL35 is the interacting protein of DDX10. In addition, we hypothesize that RPL35 is related to the E2F pathway and the immune response in CRC.

**Conclusions:**

In conclusion, provides a better understanding of the molecular mechanisms of DDX10 in CRC and provides a potential biomarker for the diagnosis and treatment of CRC.

**Supplementary Information:**

The online version contains supplementary material available at 10.1186/s12935-022-02478-1.

## Introduction

Colorectal cancer (CRC) is the most common and second most deadliest malignant tumour of the digestive system worldwide [1]. Every year, patients newly diagnosed with CRC account for 10% of newly diagnosed cancer patients worldwide. Similarly, CRC accounts for 10% of cancer-related deaths [1]. In the past 20 years, with the progress of imaging technology and treatment strategies, the five-year survival rate of CRC has risen to 65%. However, the situations of patients with advanced CRC remain dismal. The two-year survival rate of patients with advanced CRC is only approximately 20–30%. Tumour metastasis is still the main cause of death for patients with this condition [2, 3]. Therefore, reducing the proliferation and development of CRC cells has always been a difficult problem for medical workers.

In the past, research on the mechanism of tumour invasion and metastasis was mainly performed at the tissue and cellular levels. However, thanks to the rapid development of microarray and RNA sequencing (RNA-seq) technology, we began to carry out cancer research at the RNA level. In recent years, RNA helicases have attracted increasing attention from scientists. RNA helicases are enzymes that hydrolyse ATP to dissociate it from RNA and are mainly involved in RNA maturation, splicing and nuclear export processes [4]. Depending on their function and mechanism, RNA helicases are divided into different families. The RNA helicase DEAD-box (DDX) family belongs to superfamily 2, which is named because of its highly conserved amino acid sequence (Asp-Glu-Ala-Asp/His) [5]. There is a conserved core domain among DDX family members. This domain contains nine conserved motifs, including the most typical DEAD motif. These motifs endow DDX family members with ATP metabolism and RNA unwinding activities, which makes them RNA helicases. Many studies have proven that DDX family members play a crucial role in almost all steps of mRNA formation and translation [6]. Moreover, an increasing number of studies have proven that members of the DDX family are closely related to the proliferation and metastasis of tumour cells. For instance, DDX1 can affect the occurrence and metastasis of breast cancer, cervical cancer and colorectal cancer [7–11]. DDX5 is related to the progression of different tumours, such as breast cancer, colon cancer and multiple myeloma [12–20]. DDX10 promotes the proliferation of breast cancer, osteosarcoma and ovarian cancer cells [21–23]. Interestingly, we found that many members of the DDX family affect the invasion and metastasis of CRC [11, 24–28], but no study has focused on the effect of DDX10 on the prognosis of CRC. Because the structure and function of DDX family members are similar, we speculated that DDX10 is a key factor affecting the prognosis of CRC.

In our study, we analysed the expression data of CRC samples from the Gene Expression Omnibus (GEO) and The Cancer Genome Atlas (TCGA) databases to determine whether the mRNA expression of DDX10 in CRC tissues is higher than that in normal tissues. Moreover, the clinical samples we collected from The Second Affiliated Hospital of Soochow University were used for immunohistochemistry (IHC), which could detect the differences in DDX10 expression at the protein level. Subsequently, we performed cytological experiments and animal experiments to explore the role of DDX10 in CRC cells. Furthermore, we performed Gene Ontology (GO)/Kyoto Encyclopedia of Genes and Genomes (KEGG) enrichment analysis and protein–protein interaction (PPI) network analyses, which enabled us to better understand how DDX10 affects the invasion and metastasis of CRC. Finally, we predicted the interacting protein of DDX10 by LC–MS/MS and verified it by Co-IP and qPCR.

## Materials and methods

### Patients and specimens

Between January and December 2014, we collected 35 pairs of cancer and paracarcinoma tissues from patients with CRC at the Gastrointestinal Surgery Department of the Second Affiliated Hospital of Soochow University (Jiangsu Province, China). Our study was approved by the ethics committee of Soochow University. We obtained informed consent from every patient. All specimens were stored at − 80 °C.

### Immunohistochemistry

Colorectal cancer and paracarcinoma tissues were dewaxed in xylene and rehydrated through different gradients of alcohol. Then, we placed paraffin in 3% H_2_O_2_ for 15 min at 22 °C. Next, the slide was heated in citrate buffer. After washing several times with PBS (pH  = 7.2), the slide was placed into the solution with a primary antibody against DDX10 (dilution 1:50, 17857–1-AP, Proteintech, USA) at 4 °C for more than 12 h. Then, we added secondary antibody at 22 °C for 10 min. Finally, the slide was stained after the addition of 3,3′-diaminobenzidine (DAB) solution.

### Differential expression analysis

The TCGA is an open public tumour information database. We selected 50 pieces of CRC data, including that for cancer and paracarcinoma tissues, from the TCGA database to compare the mRNA expression of DDX10. The GEO database (http://www.ncbi.nlm.nih.gov/geo) [29] is an international public database that stores abundant high-throughput functional genomics data. The gene expression dataset analysed in our study was GSE74604 downloaded from the GEO. GSE74604, based on the GPL6104 platform (Illumina humanRef-8 v2.0 expression beadchip), contained data for 30 paired normal and tumour colorectal samples. We analysed the differences in gene expression data for 60 samples. Then, we also used Gene Expression Profiling Interactive Analysis 2 (GEPIA2, http://gepia.cancer-pku.cn/) [30] and ONCOMINE (https://www.oncomine.org/) to verify the results. GEPIA2 is a web tool that includes RNA-seq data for a large number of tumour and normal samples from the TCGA and Genotype-Tissue Expression (GTEx) projects. Gene expression data from ONCOMINE are freely available to the public. ONCOMINE is meant to facilitate related studies by providing genome-wide expression array data.

### Real-time PCR

Total RNA was extracted by SuPerfecTRI™ (Cat#3101-100) (Shanghai Pufei Biotech Co., Ltd). This RNA was then reverse-transcribed to cDNA by M-MLV Reverse Transcriptase (M1705) (Promega, Beijing, China). SYBR Master Mixture (TAKARA, Tokyo, JP) was used for real-time PCR. We used the expression of endogenous GAPDH as a control to take standardized quantification. The DDX10 primer sequences were as follows: forward, 5′-TTGAGGTTCTCCGAAAAGTAGG-3′ and reverse, 5′-ACATTTGGAGGTCGGTAGCAT-3′; the GAPDH primer sequences were as follows: forward, 5′-TGACTTCAACAGCGACACCCA-3′ and reverse, 5′-CCATTGCCCGTGTTCTCACA-3′; the 60S ribosomal protein L35 (RPL35) primer sequences were as follows: forward, 5′-TGACTTCAACAGCGACACCCA-3′ and reverse, 5′-GCTCCTTCCGCTGCTGCTTC-3′.

### Cell lines and lentiviral transduction

Our study used the human CRC cell lines RKO, HCT116, SW480 and LoVo, which were purchased from GeneChem (Shanghai, China). The cells were cultured in Dulbecco’s modified Eagle’s medium (DMEM; HyClone, Thermo Fisher Scientific) containing 10% foetal bovine serum (FBS; Gibco‐Invitrogen Corp.) at 37 °C with 5% CO_2_ in strict accordance with the standard. All lentiviruses used in this research were purchased from GeneChem (Shanghai, China). We transfected short hairpin RNA (shRNA) lentivirus targeting DDX10 and empty vector (controls) into HCT116 cells and RKO cells in strict accordance with the official technical instructions. The DDX10 RNAi sequence was 5′-GATGTGAGCAAGTTACCTATA-3′.

### Celigo cell growth assay

HCT116 cells and RKO cells were plated in 96-well plates (1500 cells/well). Next, we cultured the cells at 37 ℃ with 5% CO_2_. During the next five days, we counted the number of cells with a Celigo Imaging Cytometer (Nexcelom Bioscience, Lawrence, MA, USA) every day. All experiments were repeated at least three times.

### Colony formation assay and apoptosis assay

First, we collected HCT116 cells and RKO cells. Next, those cells were added to complete medium (10% FBS) and placed into 6-well plates (800 cells/well). Then, we cultured the cells in standard conditions for 8 days and changed the medium every three days. Then, they were fixed for 30–60 min with 4% paraformaldehyde. Last, we added 1000 μl/well crystal violet at room temperature. Approximately 10–20 min later, we counted the number of cells under a fluorescence microscope (Olympus).

We used annexin V-APC (eBioscience) to stain these cells to analyse apoptosis in strict accordance with official technical instructions. The cells were plated in 6-well plates (5 × 10^5^ cells/well). Then, we collected the cells and washed them with PBS. After the cells were resuspended, we added 10 μl annexin V-APC to the suspensions. Finally, we cultured the cells at 22 °C and protected them from sunlight. After 15 min, the cells were sorted by flow cytometry (C6 PLUS, BD).

### MTT assay

MTT assays were used to observe the proliferation of cells. All operations were in strict accordance with the standard. First, we placed HCT116 or RKO cells with DDX10 knockdown and negative control cells into 96-well plates (1500 cells/well) in triplicate. Then, 100 μl medium was added to every well. Finally, a microplate reader (M2009PR, Tecan Infinite) was used to quantify the colour change (490 nm) at 24 h, 48 h, 72 h, 96 h and 120 h.

### Wound-healing assay

First, we harvested HCT116 cells and RKO cells (negative control and DDX10 knockdown). Then, the cells were cultured in 96-well plates. After the confluence reached 90%, we aspirated the medium of every well and used PBS to wash the wells three times. Next, we used a pipette tip to gently draw a straight line down the wells of a 96-well plate. Finally, medium was added to every well after three washes with PBS. After 0 h, 24 h and 72 h, we used the Celigo platform to scan the plate and analyse the migration area.

### Transwell migration and invasion assays

Transwell assays were used to assess the migration and invasion abilities of HCT116 cells and RKO cells. HCT116 cells or RKO cells (1 × 10^5^) in 100 μl medium that had been cultured in serum-free medium were added to the upper chamber, and 600 μl culture medium (30% FBS) was added to the lower chamber. Then, the plate was incubated at 22 °C overnight. Finally, we dyed the cells on the lower surface of the membrane to analyse cell migration. The Transwell invasion assay was carried out as described above, but Matrigel was added to the bottom of the upper chamber.

### Animal studies

The animal experiment of our study used 5-week-old female BALB/c nude mice (GemPharmatech, Co., Ltd., China) weighing between 16 and 19 g. Twenty nude mice were randomly divided into two groups with 10 mice in each group. Two groups (negative control and DDX10 knockdown) of HCT116 cells (stably expressing luciferase) (2 × 10^6^ cells/100 μl) were injected into the mice via the tail vein. Then, we used an in vivo imaging system (Lumina LT, Perkin Elmer, USA) to observe tumour metastasis every week. We anaesthetized the mice before performing imaging monitoring. First, the mice were intraperitoneally injected with D-luciferin (15 mg/ml) at a concentration of 10 μl/g. After 15 min, the mice were anaesthetized by intraperitoneal injection of 0.7% pentobarbital sodium at a concentration of 10 μl/g. After a few minutes, the mice were anaesthetized and placed under bioluminescence imaging for imaging monitoring. All mice were cultured in a specific-pathogen-free (SPF) culture environment. At the same time, we also measured and recorded the tumour size.

### Mass spectrometry and protein identification

Briefly, we used a scraper to harvest RKO cells that stably expressed Flag-DDX10 and lysed the cells with lysis buffer. The reference dosage for Co-IP was 400 µl FLAG beads to collect 15,000 μg of protein. First, we used weak lysis buffer to wash the beads four times. Then, we carried out SDS-PAGE and placed the gel into Coomassie brilliant blue R250 solution for staining. Next, we collected the protein bands and used LC–MS for analysis. The original graph file (.raw) exported from Q Exactive was changed into a “.mgf ” file by Proteome Discoverer 2.1 (Thermo Fisher Scientific), and then we delivered these data to the MASCOT2.6 server for database searching by built-in facilities. Then, we received the search files (.dat) that had been processed from the MASCOT server. Finally, we selected candidate peptides according to the standard of FDR  < 0.01. The protein database used in this analysis was UniProt_HomoSapiens_20386_20180905.

### LinkedOmics

LinkedOmics (http://www.linkedomics.org/) [31] is an unique platform for biologists and clinicians to access, analyze and compare cancer multi-omics data within and across tumor types. In our study, we used the “colorectal adenocarcinoma (TCGA_COADREAD)” dataset to analyse the correlation between E2F family and DDX10 or RPL35.

### Co-immunoprecipitation assay

First, we collected RKO cells that overexpressed DDX10 after washing them three times with PBS. Then, these cells were added to precooled cell lysis buffer. Next, the final cell lysates were sonicated and centrifuged at 14,000 rpm for 10 min. The lysates were mixed with preblocked Flag beads (A2220, Sigma) at 4 °C overnight. After Co-IP, the Flag beads were washed as follows. First, we washed with lysis buffer three times. Then, we added 6 × loading buffer and left the samples at room temperature for 10 min. Finally, the cell lysates were mixed with 5 × sodium dodecyl sulfate (SDS) loading buffer and then boiled for 10 min. Proteins were eluted from the beads and separated by western blotting.

### Western blot analysis

We used a 15% SDS-PAGE gel to isolate proteins, and the proteins were transferred to a PVDF membrane. The primary antibodies used in our study were mouse anti-GAPDH (1:5000; Santa Cruz, USA), rabbit anti-RPL35 (1:100; ABclonal, China), rabbit anti-DDX10 (1:300; Proteintech, USA), mouse anti-FLAG (1:2,000; Sigma, USA), rabbit anti-RPL18 (1:100; ABclonal, China), rabbit anti-HNRNPU (1:100; ABclonal, China), rabbit anti-NCL (1:100; ABclonal, China), rabbit anti-PRMT5 (1:100; ABclonal, China), rabbit anti-E2F1 (1:100; ABclonal, China), rabbit anti-E2F4 (1:100; ABclonal, China), rabbit anti-E2F8 (1:100; ABclonal, China), goat anti-rabbit IgG (1:2,000; CST, USA), and goat anti-mouse IgG (1:2,000; CST, USA).

### Enrichment analyses and PPI network construction

GO and KEGG analyses are important bioinformatics tools for annotating genes and researching gene functions, biological processes and signalling pathways [32–34]. In our study, we analysed the genes that were downregulated by DDX10 antibody using R version 4.0.5. The PPI network for these genes was predicted using the Search Tool for the Retrieval of Interacting Genes (STRING; https://string-db.org/) online database [35].

### Gene set enrichment analysis (GSEA)

The RNA-seq data from the COAD and READ datasets were obtained from the TCGA portal (https://portal.gdc.cancer.gov/). Then, we divided the TCGA samples into two groups according to the expression of DDX10 and analysed these two groups of data by GSEA. Finally, we screened the results with the criterion of normalized enrichment score (NES) ≥ 1.0 and adjusted P value < 0.25.

### Tumour immune estimation resource (TIMER) database analysis

TIMER (https://cistrome.shinyapps.io/timer/) [36, 37] is an interactive website offering comprehensive analysis of immune infiltration among different types of cancer. TIMER uses various immune deconvolution methods to calculate the immune infiltrate abundances. Moreover, the subscriber can obtain high-quality figures. In this work, we searched “RPL35; COAD” in the “gene module” and performed an analysis of immune infiltration.

### Statistical analysis

We used Prism version 8.0 software (GraphPad Software Inc.) to analyse all the data in this study. Unpaired t tests were used to compare two groups, and one-way ANOVA was used to compare multiple groups. The screening criteria for all data was P value  < 0.05. All of the trials were repeated at least three times.

## Results

### DDX10 expression is upregulated in CRC tissue and CRC cell lines

**Fig. 1:**
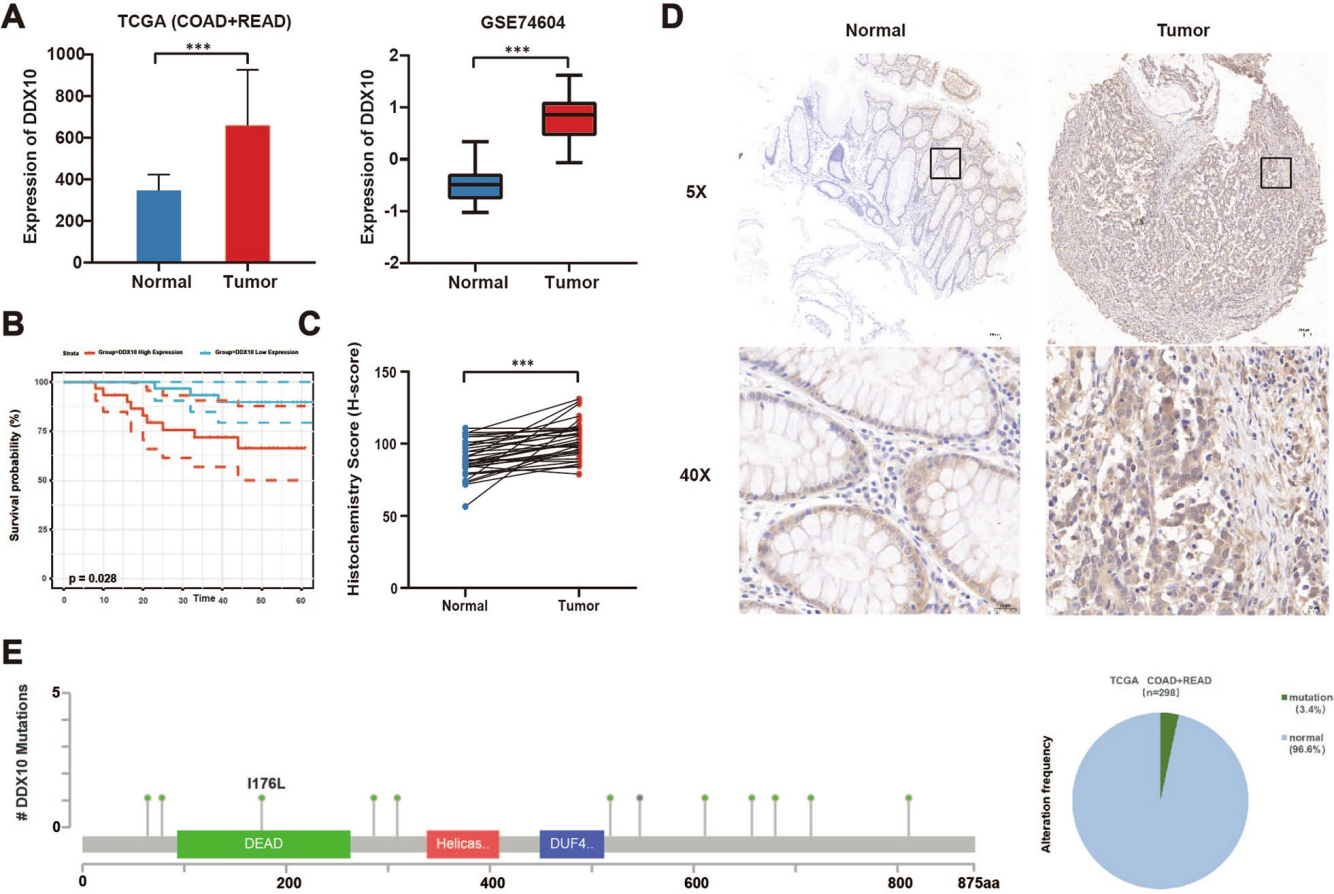
Clinical significance of DDX10 expression in colorectal cancer (CRC). **A** mRNA expression of DDX10 in CRC tissues and normal colon tissues in The Cancer Genome Atlas (TCGA) database and Gene Expression Omnibus (GEO) database; **B** Kaplan–Meier disease-free survival curves of patients with CRC according to DDX10 expression in GSE17537; **C** Immunohistochemical results of clinical samples (n  = 35, H-score, P  < 0.05); **D** Immunohistochemical staining for DDX10 in CRC tissues and normal tissues; **E** Positions and frequency of mutations in DDX10 among CRC cases in TCGA dataset. Number of mutations was observed in twelve cases and frequency was 3.4%

**Fig. 2:**
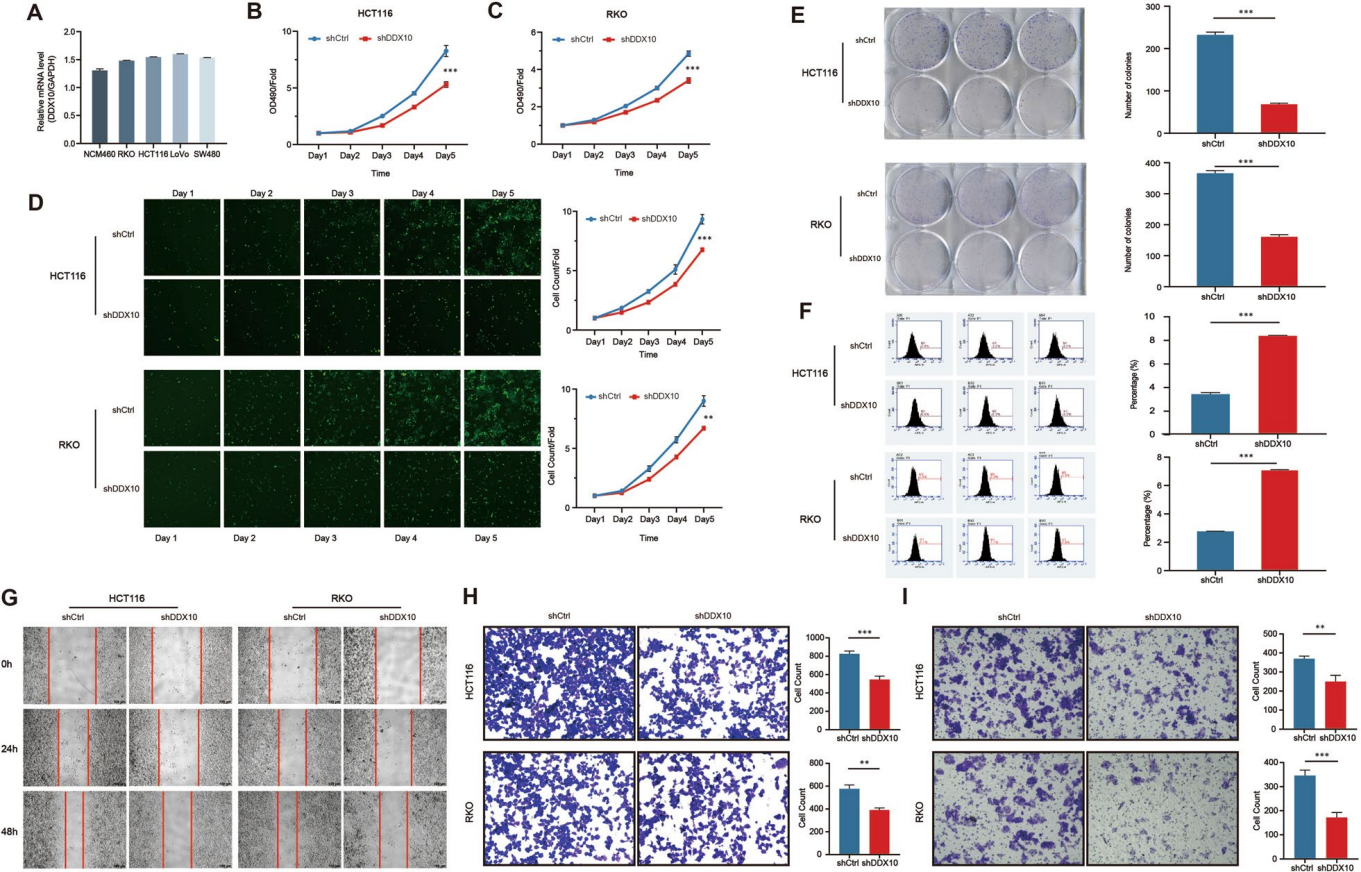
DDX10 promoted colorectal cancer cell growth and metastasis in vitro. **A** Expression of DDX10 in several colorectal cancer cell lines by q-PCR; MTT cell proliferation assay was applied. Cell growth rate was significantly suppressed by shDDX10 (**B**) HCT116 cells (P  < 0.001) (**C**) RKO cells (P  < 0.001); **D** Fluorescence images of HCT116 (P < 0.001) and RKO cells (P < 0.01) were measured via Celigo high-content screening assay. Upper panel was the representative images and lower panel was the growth curve; **E** Colony formation assays using DDX10‐knockdown HCT116 and RKO cells (P < 0.001); **F** Cell apoptosis using DDX10‐knockdown HCT116 and RKO cells (P < 0.001); G. Wound-healing assay using DDX10-knockdown HCT116 and RKO cells; H. Transwell migration assay using DDX10-knockdown HCT116 (P < 0.001) and RKO cells (P < 0.01); I. Transwell invasion assays using DDX10-knockdown HCT116 (P < 0.01) and RKO cells (P < 0.001)

We analysed the gene expression of 50 paired cancer and paracarcinoma tissues selected from the TCGA database and found that the mRNA expression of DDX10 in CRC tissues was evidently higher than that in paracarcinoma tissues (Fig. 1A). By analysing the mRNA expression data of 30 pairs of samples from the GEO database (GSE74604), we obtained a list of differentially expressed genes (Additional file 1: Fig. S1A); the mRNA expression of DDX10 in CRC was evidently higher than that in paracarcinoma tissues (Fig. 1A). Next, we used online analytics websites to retrieve data for DDX10. The results of the GEPIA2 and ONCOMINE database analysis also verified that the mRNA expression of DDX10 was significantly different between tumour and normal tissues (Additional file 1: Fig. S1B-F; Table 1). All these database results showed that the mRNA expression of DDX10 was upregulated in CRC tissue. As expected, compared with that in paracarcinoma tissues, the protein expression of DDX10 in tumour tissues was significantly elevated (Fig. 1C, D). Survival analyses of disease-free survival in COAD revealed that the expression of DDX10 is associated with the prognosis of patients (Fig. 1B). The rate of mutations in DDX10 was 3.4% in the TCGA (Fig. 1E). Furthermore, compared with normal intestinal epithelial cells (NCM460), higher expression of DDX10 was shown in many CRC cell lines (LoVo, HCT116, RKO, SW480) with GAPDH as an internal reference gene (Fig. 2A).

**Table 1:** The expression levels of DDX10 in different types of tumor and normal tissues (UALCAN)

Type of tumor	Maximum	Minimum	Median	P value
BLAC				
Tumor	26.716	1.093	12.897	4.598200E−03
Normal	15.437	7.483	12.837	
CHOL				
Tumor	16.393	4.659	10.713	1.700750E−12
Normal	4.804	2.332	3.305	
COAD				
Tumor	33.192	7.078	18.391	1.624478E−12
Normal	11.336	3.544	7.439	
ESCA				
Tumor	38.759	4.748	16.977	5.969599E−07
Normal	15.079	4.143	6.562	
LIHC				
Tumor	15.539	0.842	6.313	1.624367E−12
Normal	5.35	1.06	3.834	
READ				
Tumor	30.994	1.888	17.34	1.086199E−08
Normal	10.149	7.718	8.57	
STAD				
Tumor	31.934	4.648	14.939	< 1E−12
Normal	12.23	2.641	6.215	

*BLCA* bladder urothelial carcinoma; *STAD* stomach adenocarcinoma; *COAD* colon adenocarcinoma; *READ* rectum adenocarcinoma; *CHOL* cholangiocarcinoma; *LIHC* liver hepatocellular carcinoma; *ESCA* esophageal carcinoma

### DDX10 promotes CRC cell proliferation and metastasis in vitro

Analysis of the relationship between the mRNA expression of DDX10 and the staging of CRC in the ONCOMINE database indicated that DDX10 expression in tumour tissues in M1 stage (Additional file 1: Fig. S1G) or Dukes’ D stage (Additional file 1: Fig. S1H) was higher than that in tissues in M0 or Dukes’ A stage.

To clarify the effect of DDX10 on the invasion and metastasis of CRC cells, LV-shDDX10 HCT116 and RKO cells were generated, and the influence on DDX10 expression was proven by qRT-PCR (Additional file 2: Fig. S2A). MTT (Fig. 2B, C) and cell counting assays (Fig. 2D) showed that the decrease in DDX10 expression significantly suppressed the growth of HCT116 and RKO cells. Next, the results of the colony formation test revealed that the colony number of the shDDX10 cell group were evidently reduced compared with that of the shCtrl cell group (Fig. 2E). Then, we assessed cell apoptosis by fluorescence-activated cell sorting (FACS). Five days after shRNA lentivirus infection, the number of apoptotic HCT116 and RKO cells had increased markedly in the experimental group (shDDX10) (Fig. 2F). These outcomes suggested that HCT116 and RKO cell proliferation was suppressed due to the decreased expression of DDX10. Finally, the outcomes of the wound-healing assay and Transwell migration assay indicated that decreased expression of DDX10 could significantly reduce the wound-healing rate and the number of metastatic HCT116 and RKO cells (Fig. 2G, H). Transwell invasion assays also showed that, compared with control cells, fewer HCT116 and RKO cells with extremely low expression of DDX10 passed through the cell-permeable membrane (Fig. 2I). This result showed that DDX10 could enhance the invasiveness of CRC cells. Based on the above experimental results, our study indicates that DDX10 potentially has the ability to promote the development and metastasis of CRC.

### DDX10 promotes colon cancer cell metastasis in vivo

**Fig. 3:**
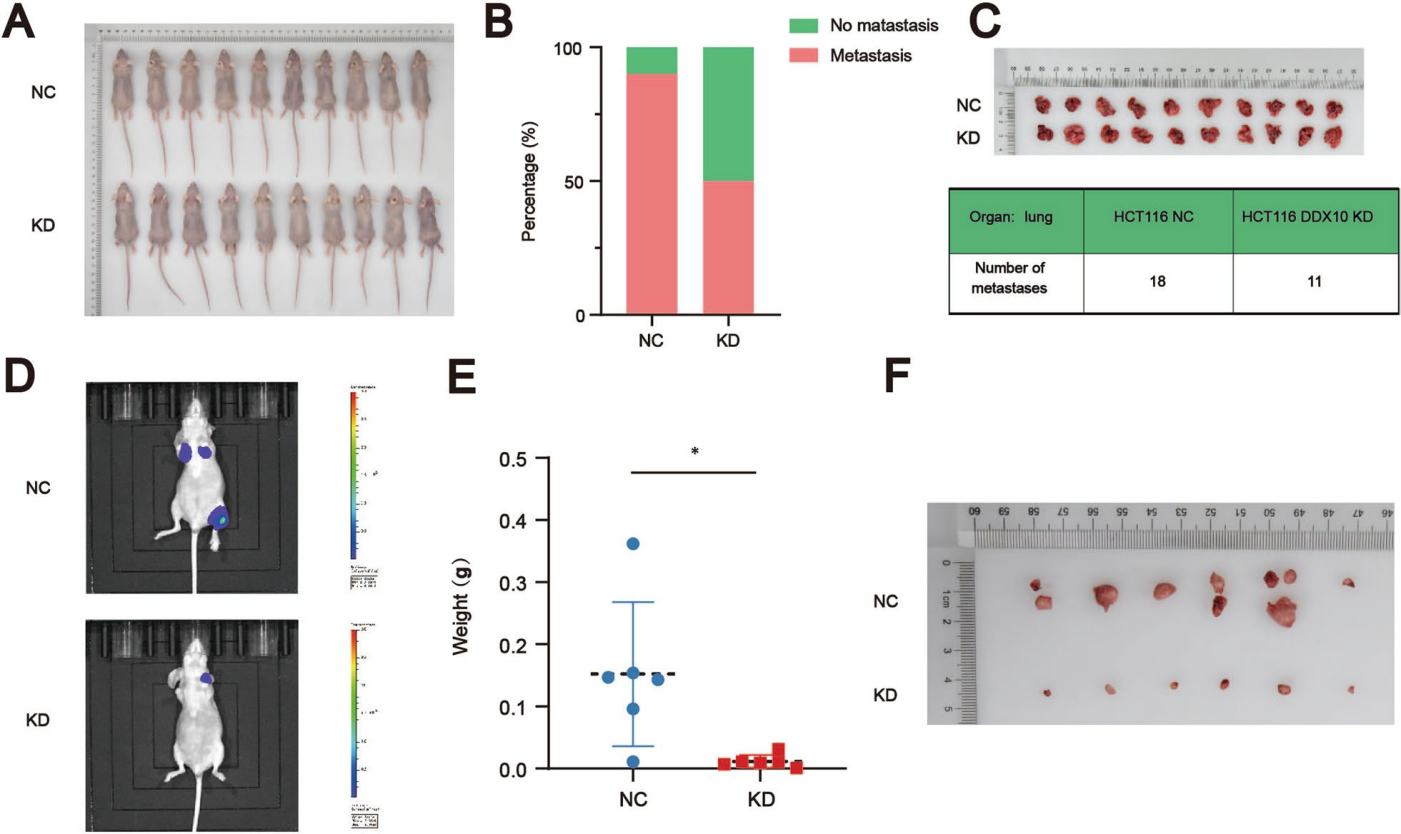
DDX10 promoted colorectal cancer cell metastasis in vivo. **A** Twenty nude mice were used in our study; **B** The metastasis rates of the negative control group (90%) and knockdown group (50%); **C** The number of metastatic tumors of CRC; **D** Bioluminescence imaging (BLI) of mice after tail vein injection; **E** The weight of metastatic tumors of CRC (P < 0.05); **F** Tumor metastasis model of CRC after tail vein injection

To clarify the function of DDX10 in metastasis in vivo, we constructed a metastasis model of CRC in nude mice (Fig. 3A). In our study, colorectal cancer metastasis was found only in the lung. The number and weight of lung metastatic nodules in mice were observed 37 days after tail vein injection. The final results showed that the number of lung metastatic nodules was lower (Fig. 3C) (the metastasis rates of the negative control group and knockdown group were 90% and 50%, respectively) (Fig. 3B), and the total weight of all metastatic nodules in mice injected with HCT116/LV-shDDX10 cells was higher than that of all metastatic nodules in mice injected with HCT116/LV-shCtrl cells (Fig. 3E, F). In addition, the total fluorescence intensity in the region was greater in mice that were injected with HCT116/LV-shDDX10 cells than in those injected with HCT116/LV-shCtrl cells (Fig. 3D). Therefore, this study demonstrated that DDX10 has roles in promoting CRC growth and progression in vivo.

### DDX10 expression is positively correlated with the cell cycle and RNA splicing

**Fig. 4:**
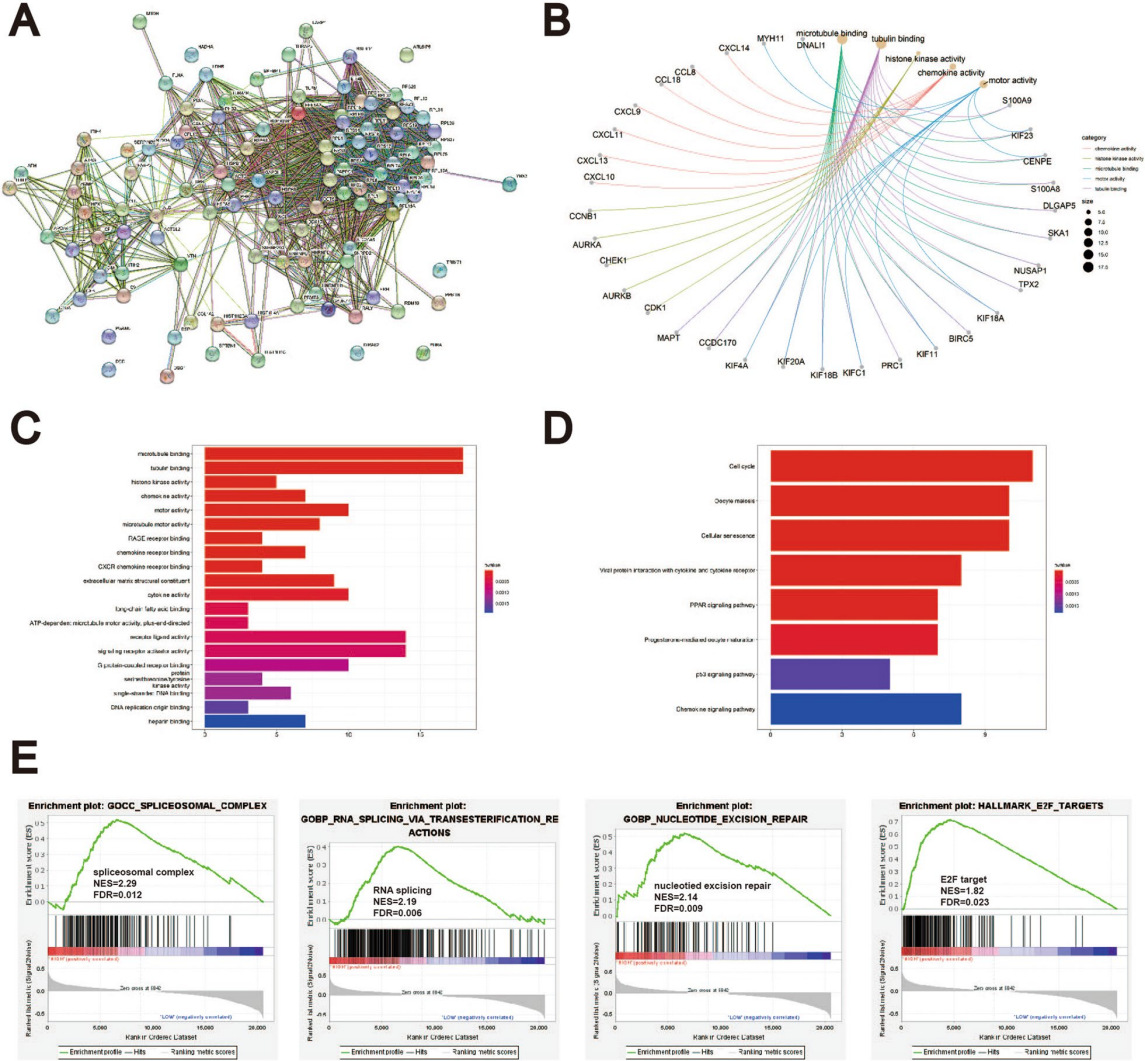
PPI network construction and Enrichment analysis **A** Protein–protein interaction network of DDX10 constructed by STRING; **B**–**D**. GO functional enrichment analysis and KEGG pathway enrichment analysis by R (version 4.0.5). Overexpression of DDX10 was positively correlated with microtubule binding, cell cycle; **E** GSEA analysis was performed using TCGA dataset. DDX10 is closely related to RNA splicing and E2F targets

The PPI network among these proteins interacting with DDX10 was predicted by STRING tools. We found 110 proteins interacting with DDX10 in the suspension of the Co-IP assay by LC–MS. These 110 proteins were involved in a complex PPI network with a total of 108 nodes and 1,181 edges (Fig. 4A). In this network, the average local clustering coefficient was 0.649, and the PPI enrichment P value was  < 1.0e−16.

To explore the mechanism by which DDX10 regulates the occurrence and development of CRC, these 110 proteins were used for GO/KEGG enrichment analysis. The results indicated that DDX10 was positively correlated with microtubule binding, the cell cycle and RNA splicing (Fig. 4B–E).

### DDX10 may alternatively splice the mRNA of RPL35 and then work through the E2F pathway

**Fig. 5:**
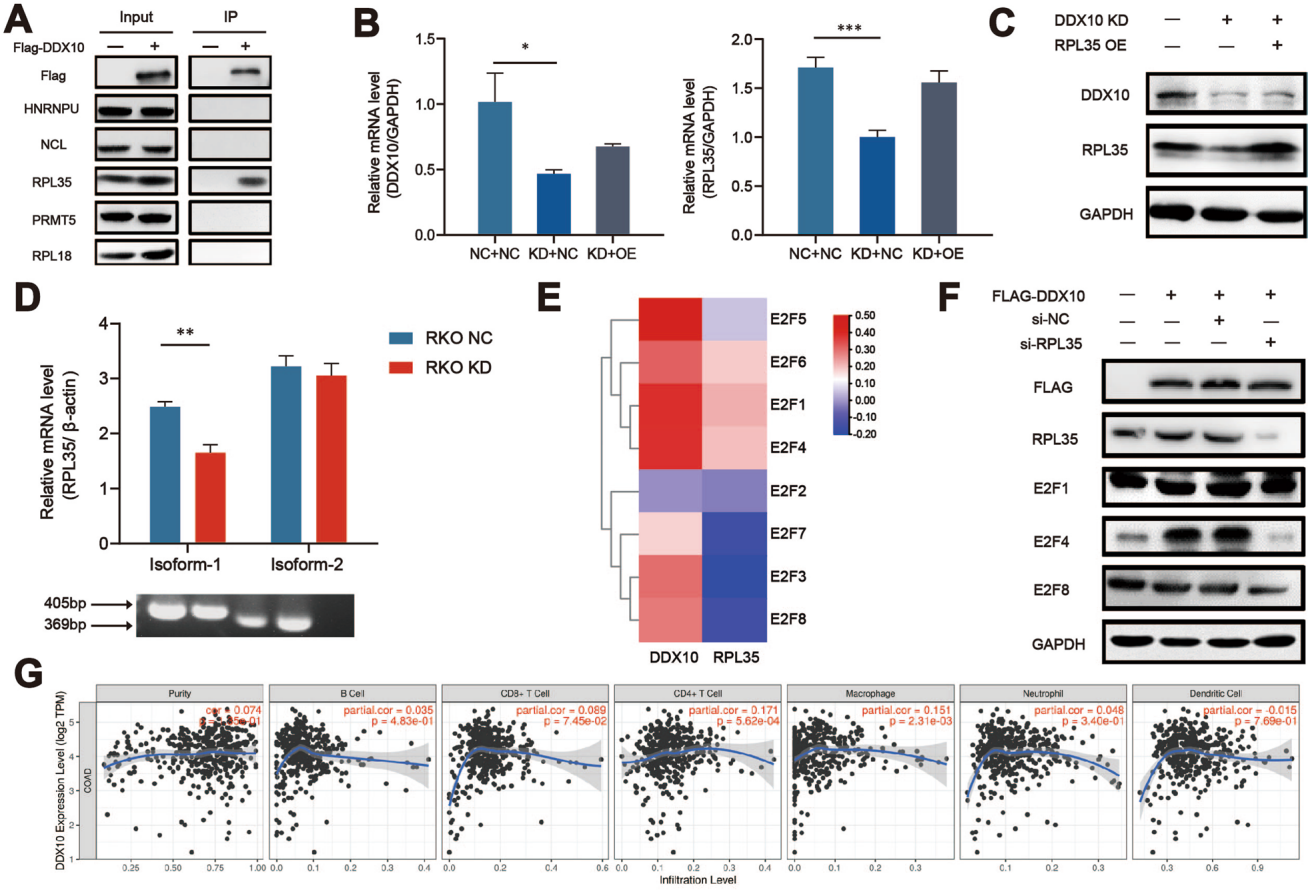
DDX10 alternatively splices the mRNA of RPL35 then work through E2F pathway or immune response of CRC **A** RPL35 interacts with DDX10 through the result of Co-IP; **B** Overexpression of DDX10 obviously reduced the expression of RPL35 at mRNA level via q-PCR; **C** Overexpression of DDX10 obviously reduced the expression of RPL35 at protein level; **D** The expression of two mRNA isoforms of RPL35 in normal HCT116 cells and DDX10 knockdown HCT116 cells by q-PCR; **E** DDX10 and RPL35 are closely related to E2F1 and E2F4 from the data of LinkedOmics; **F** DDX10 interacts with RPL35 to regulate E2F1 and E2F4 to regulate colorectal cancer; **G** The relationship between DDX10 and immune cells that infiltrate the tumor tissue in COAD

To better study the biological functions and pathways in which DDX10 participates, we searched for distinctive proteins that interact with DDX10. Cell extracts were applied to LC–MS/MS analysis, and the output is shown in the Additional file 3: Table S1. A large number of Co-IP assays and western blot experiments were used to verify the interaction between the proteins selected by LC–MS/MS and DDX10 (Fig. 5A). Eventually, we found that RPL35 is very likely to interact with DDX10. As expected, the q-PCR and western blot results showed that the decreased expression of DDX10 obviously reduced the expression of RPL35, which verified our conclusion (Fig. 5B, C). At the same, we detected the expression of two mRNA isoforms of RPL35 in normal HCT116 cells and DDX10 knockdown HCT116 cells. Amazingly, the absence of DDX10 did change the two isoforms of RPL35 (Fig. 5D). E2F transcription factors (E2Fs) are the most important genes regulating the progression of CRC. The outcomes of LinkedOmics indicated that DDX10 and RPL35 are closely related to E2F1, E2F4 and E2F5 in CRC (Fig. 5E). In addition, from the GSEA results, we observed that the expression of DDX10 was closely related to E2F targets (Fig. 4E). Therefore, we use western blot to verify that E2Fs pathway is indeed the downstream pathway of DDX10 and RPL35 (Fig. 5F). Considering the above results, we propose that DDX10 may regulate the occurrence and development of CRC through the E2F pathway after alternatively splicing RPL35 mRNA.

### DDX10 is most likely related to the immune response of CRC

The TIMER results indicated that the expression of DDX10 is closely related to the infiltration of CD4 + T cells, CD8 + T cells and macrophages in COAD (Fig. 5G). Therefore, we surmised that DDX10 influences the occurrence and development of CRC by regulating immune cell infiltration, which would be an interesting research direction.

## Discussion

In recent years, CRC has become the second leading cause of death in cancer patients. The ultimate cause of death in CRC patients is usually tumour metastasis. Therefore, it is very important to explore the mechanism underlying the metastasis of CRC cells. Many DDX family members, including DDX1, DDX3, DDX5, DDX17, DDX27 and DDX56, have been confirmed to be closely related to CRC [11, 24, 28, 38–40]. Moreover, through the research of Patrick Linder et al. [5] we know that the members of the DDX family are similar in structure and function. Hence, we reasonably speculated that DDX10 plays a key role in the invasion and metastasis of CRC cells.

DDX10 is a member of the DDX family that is characterized by the presence of an Asp-Glu-Ala-Asp (DEAD) motif, and its main function is to mediate the biogenesis of ribosomes [41]. However, it was not until the late 1990s that DDX10 attracted the attention of biologists [41, 42]. Our pancancer analysis results showed that DDX10 is highly expressed in a variety of cancer tissues; therefore, DDX10 is likely to be a key gene in many cancers. Previous studies have shown that the fusion of NUP98 and DDX10 may lead to leukaemia [43]. Through gene rearrangement studies, Dr. Jiao confirmed that DDX10 is a potential oncogene affecting the growth and proliferation of breast cancer cells [21]. Recently, it was confirmed that the expression of DDX10 is significantly higher in osteosarcoma patients [22]. Interestingly, DDX10 plays an antitumour role in the development of ovarian cancer. Studies have shown that decreased expression of DDX10 promotes the proliferation of ovarian cancer through the Akt/NF-kB pathway [23]. In our study, we first reported that DDX10 was upregulated in CRC and was closely associated with CRC clinical stage and prognosis. To the best of our knowledge, this is the first study on the function of DDX10 as a carcinogenic driver and prognostic biomarker for CRC.

In our study, the data from multiple databases were used to verify that the expression of DDX10 in tumour tissues was evidently higher than that in normal tissues, and the difference was also obvious in tumour-node-metastasis (TNM) stage and Dukes’ stage. Then, we used a lentiviral transfection method to knockdown and overexpress DDX10 to carry out cytological experiments and to construct a tumour metastasis model in mice, which helped us explore the effect of DDX10 on the growth, invasion and metastasis of CRC cells. These results showed that the growth of CRC cells was significantly inhibited after DDX10 knockdown and that the invasion ability was also significantly reduced. The decrease in the number and total weight of metastatic tumours in mice injected with shDDX10-CRC cells also fully demonstrated that the expression of DDX10 promoted the migration of CRC. The above results strongly suggest that DDX10 is a key gene regulating the invasion and metastasis of CRC and that DDX10 could be used as a new target for CRC treatment.

To better comprehend the mechanism of DDX10 in CRC, we carried out LC–MS/MS analysis and employed Co-IP, q-PCR and western blotting to verify the interaction. Fortunately, we found that DDX10 and RPL35 were strongly coexpressed. RPL35 encodes a ribosomal protein that is a component of the 60 s subunit. The 60 s subunit and 40 s subunit make up ribosomes that catalyse protein synthesis. RPL35 belongs to the L29 family of ribosomal proteins. These proteins of this family are usually associated with the activation of oncogenes and anti-oncogenes. However, there are few studies on RPL35. At present, studies on the identified diseases related to RPL35 mainly focus on Diamond-Blackfan anaemia (DBA) [44]. In addition, one study speculated that the expression of RPL35 could predict lymph node metastasis in patients with early-stage cervical carcinoma [45]. A recent study reported that lncNB1 promotes neuroblastoma tumorigenesis by interacting with RPL35. This study also identified that RPL35 is a key factor for promoting E2F1 protein synthesis [46]. E2Fs are a classic group that regulate the occurrence of cancer and the cell cycle. Moreover, the GSEA results showed that the high expression of DDX10 is closely related to the cell cycle and E2F targets. Therefore, we speculated whether the E2F pathway is a key pathway in regulating CRC after DDX10 alternatively splices the mRNA of RPL35. To confirm our conjecture, we observed the relationship between DDX10, RPL35 and E2Fs in LinkedOmics. As expected, both DDX10 and RPL35 were closely related to E2F1, E2F2, E2F3, E2F4 and E2F5. Consequently, we believe that the E2F pathway is very likely to play an important role in the DDX10-RPL35-CRC regulatory pathway. Alternative splicing of mRNA is an important way to regulate gene expression in cancer development. As an RNA helicase, DDX10 is likely to participate in the splicing of the mRNA of RPL35. As we expected, high expression of DDX10 increased the incidence of AT (Alternate terminator) in mRNA of RPL35. Interestingly, through the TIMER database, we found that there was a strong correlation between the expression of DDX10 and the number of infiltrating immune cells, such as CD4 + T cells, CD8 + T cells and macrophages, in COAD. Based on this discovery, we hypothesize that DDX10 may mediate the immune response in the development of CRC, which must be of great significance for the immunotherapy of CRC.

## Conclusion

In summary, we determined that DDX10 is a novel oncogene that affects the mRNA expression of RPL35. Moreover, we speculate that RPL35 may be related to the E2F pathway and immune response in CRC. Our study showed that DDX10 is a potential therapeutic target for CRC and could have important significance for clinical research on CRC.

## Supplementary Information

**Additional file 1.** DDX10 is a critical biomarker of colorectal cancer.

**Additional file 2.** Biological mechanism of DDX10.

**Additional file 3: Table S1.** The proteins selected by LC-MS/MS

## Data Availability

The data used in the current study are available from the corresponding author on reasonable request. The TCGA data of the gene expression RNAseq were from the database UCSC Xena. The GEO dataset (GSE74604) of the gene expression RNAseq were from GEO database.
