# Supplementary figures and images for: DDX10 promotes the proliferation and metastasis of colorectal cancer cells via splicing RPL35
Source: Cancer Cell Int. 2022 Feb 2;22:58. doi: 10.1186/s12935-022-02478-1 (PMC8812018; doi:10.1186/s12935-022-02478-1)

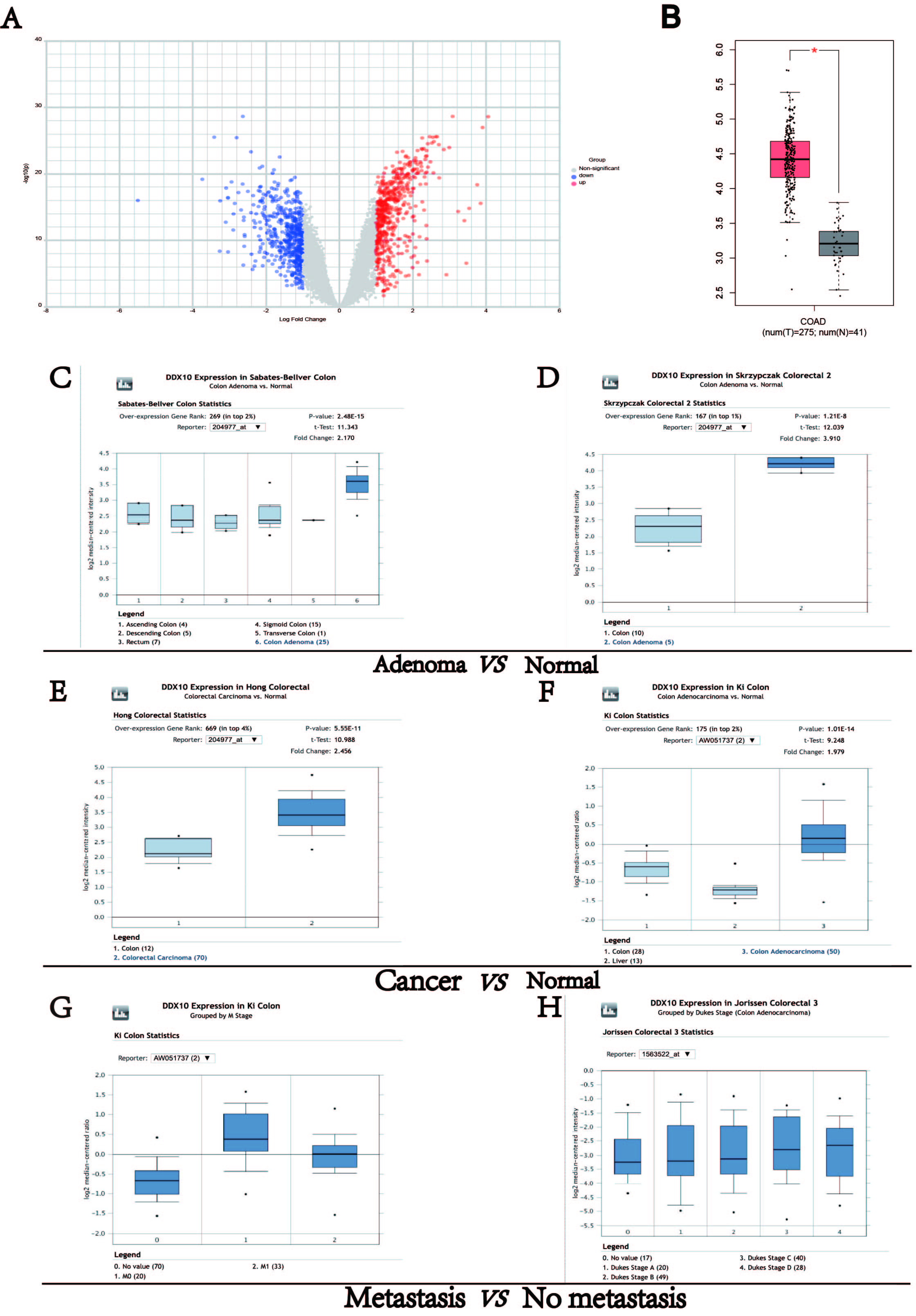

Supplement: Supplementary file 1 — Additional file 1. DDX10 is a critical biomarker of colorectal cancer. [file 12935_2022_2478_MOESM1_ESM.jpg]

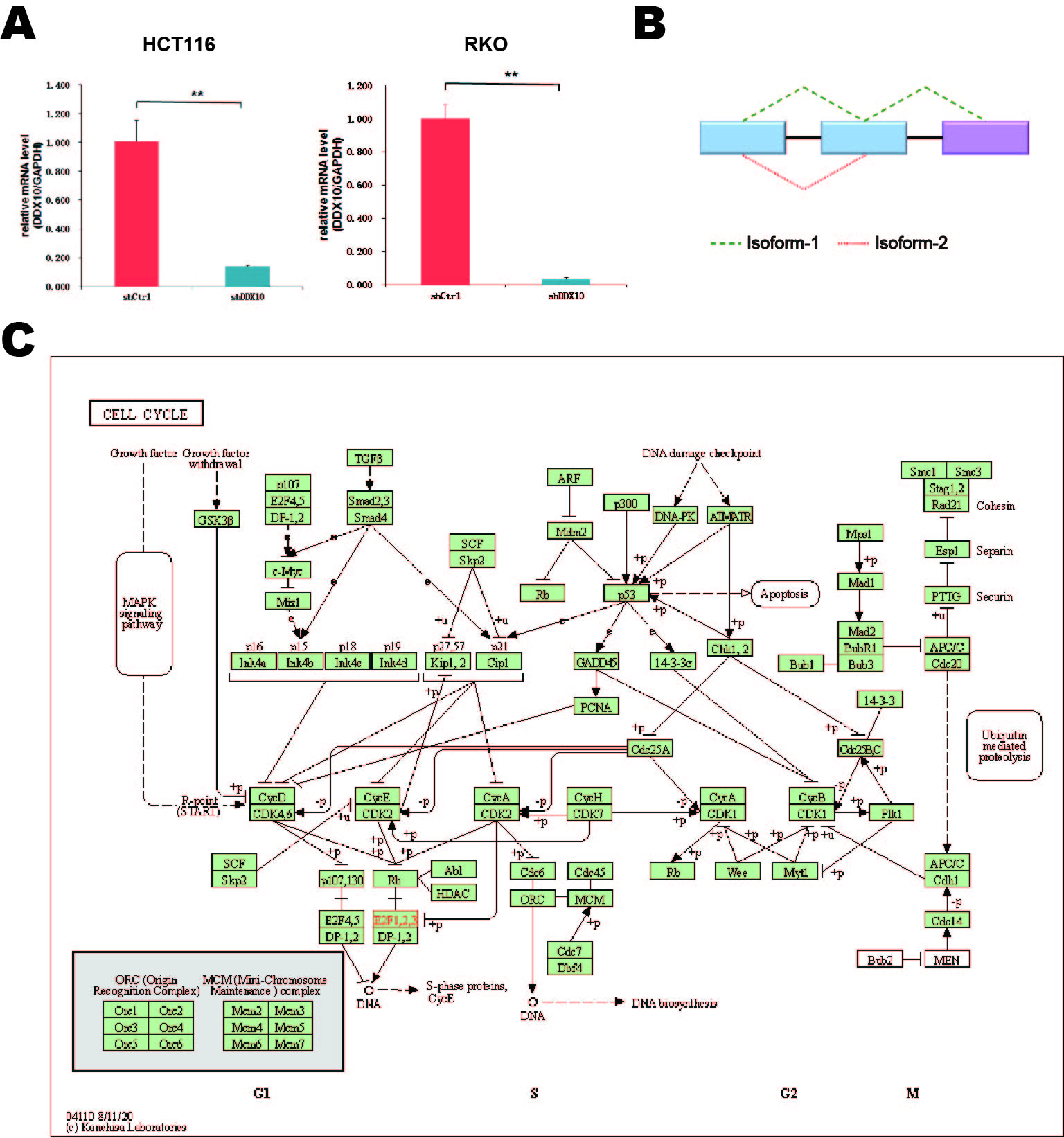

Supplement: Supplementary file 2 — Additional file 2. Biological mechanism of DDX10. [file 12935_2022_2478_MOESM2_ESM.jpg]
