# Supplementary material for: DDX10 promotes the proliferation and metastasis of colorectal cancer cells via splicing RPL35
Source: Cancer Cell Int. 2022 Feb 2;22:58. doi: 10.1186/s12935-022-02478-1 (PMC8812018; doi:10.1186/s12935-022-02478-1)
Supplement: Supplementary file 3 — Additional file 3: Table S1. The proteins selected by LC-MS/MS [file 12935_2022_2478_MOESM3_ESM.docx]

Extended data Table 1 | The proteins selected by LC-MS/MS

| **Gene** | **Gene** | **Gene** | **Gene** | **Gene** | **Gene** | **Gene** | **Gene** | **Gene** |
| --- | --- | --- | --- | --- | --- | --- | --- | --- |
| YBX3 | SRFBP1 | PHB | HIST1H1C | EIF4B | C1QA | RPL8 | RPL14 | RPS2 |
| VTN | ALB | DDX10 | HNRNPU | ERH | COL1A2 | RPL7A | RPL13 | RPS19 |
| GC | SERPINB3 | PABPC1 | HNRNPL | HSPA5 | CFL1 | RPL6 | RPL12 | RPS18 |
| SNRNP200 | PGAM5 | NCL | HNRNPH1 | TUFM | CLU | RPL4 | RPL11 | RPS14 |
| TUBB | RALY | WDR77 | HPX | EEF1A2 | CP | RPL35 | RPL10A | RPS13 |
| TUBA1B | RBM10 | LDHB | HSP90AB1 | TRIM71 | CANX | RPL34 | RPL10 | RPS11 |
| HADHA | RSL1D1 | LARP1 | HSPB1 | DSP | ACTBL2 | RPL32 | RPLP0 |  |
| PURA | PPM1B | ITIH4 | HSPA8 | DSG1 | APOA4 | RPL3 | RPS9 |  |
| THRAP3 | MTDH | ITIH2 | DIRAS2 | DCD | A1BG | RPL28 | RPS4Y1 |  |
| CCT5 | PDIA3 | ITIH1 | GAPDH | CFB | AFM | RPL26 | RPS3A |  |
| HSPA9 | PRMT5 | IGKV2-40 | ALDOA | C9 | ARL6IP6 | RPL19 | RPS27 |  |
| SPTBN1 | AMBP | HIST1H4A | FLNA | C4A | SLC25A5 | RPL18A | RPS26 |  |
| SNRPD2 | PHB2 | HIST1H2BA | FABP5 | C3 | ACTB | RPL18 | RPS23 |  |
